# Composition of essential oils of four *Hedychium* species from Vietnam

**DOI:** 10.1186/s13065-014-0054-3

**Published:** 2014-08-28

**Authors:** Bui Van Thanh, Do N Dai, Tran D Thang, Nguyen Q Binh, Luu D Ngoc Anh, Isiaka A Ogunwande

**Affiliations:** Institute of Ecology and Biological Resources, Vietnam Academy of Science and Technology, 18-Hoang Quoc Viet, Cau Giay, Ha Noi, Vietnam; Faculty of Agriculture, Forestry and Fishery, Nghe An College of Economics, 51-Ly Tu Trong, Vinh City, Nghe An Province Vietnam; Faculty of Chemistry, Vinh University, 182-Le Duan, Vinh City, Nghe An Province Vietnam; Vietnam National Museum of Nature, Vietnam Academy of Science and Technology, 18-Hoang Quoc Viet, Cau Giay, Ha Noi, Vietnam; Natural Products Research Unit, Department of Chemistry, Faculty of Science, Lagos State University, Badagry Expressway Ojo, P. M. B. 0001, Lasu Post Office, Ojo, Lagos, Nigeria

**Keywords:** *Hedychium*, Essential oil composition, GC-MS, Monoterpenes, Sesquiterpenes

## Abstract

**Background:**

Vietnam is a country blessed with many medicinal plants widely used as food and for medicinal purposes, and they contain a host of active substances that contribute to health. However, the analysis of chemical constituents of these plant species has not been subject of literature discussion.

**Results:**

In this study, the chemical compositions of essential oils of four *Hedychium* species, obtained by hydrodistillation, were determined by means of gas chromatography-flame ionization detector (GC-FID) and gas chromatography–mass spectrometry (GC-MS) techniques. Individually, *α*-pinene (52.5%) and β-pinene (31.8%) were present in the leaf oil of *Hedychium stenopetalum* Lodd., while linalool (45.2%), (*E*)-nerolidol (8.7%) and *α*-pinene (5.0%) were identified in the root. The leaf of *Hedychium coronarium* J. König was characterized by *β*-pinene (20.0%), linalool (15.8%), 1,8-cineole (10.7%), *α*-pinene (10.1%) and *α*-terpineol (8.6%); while *β*-pinene (23.6%), *α*-humulene (17.1%) and *β*-caryophyllene (13.0%) were identified in the root. *Hedychium flavum* Roxb., gave oil whose major compounds were *β*-pinene (22.5%), *α*-humulene (15.7%) and *β*-caryophyllene (10.4%) in the leaf; *α*-humulene (18.9%), *β*-caryophyllene (11.8%) and *β*-pinene (11.2%) in the stem, as well as *β*-pinene (21.8%), linalool (17.5%) and 1,8-cineole (13.5%) in the root. The main constituents of *Hedychium ellipticum* Buch.-Ham. ex Smith were (*E*)-nerolidol (15.9%), *β*-pinene (11.8%) and bornyl acetate (9.2%) in the leaf with 1,8-cineole (40.8%), *α*-pinene (18.3%) and *β*-pinene (11.0%) occurring in the root.

**Conclusions:**

Ubiquitous monoterpenes and sesquiterpenes were identified as characteristic markers for *Hedychium* species. This work is of great importance for the evaluation of *Hedychium* essential oils grown in Vietnam.

**Electronic supplementary material:**

The online version of this article (doi:10.1186/s13065-014-0054-3) contains supplementary material, which is available to authorized users.

## Background

In continuation of our research aimed at the isolation and characterization of volatile constituents of Vietnamese flora [1], we report herein compounds identified in four *Hedychium* species. The genus *Hedychium* consists of about 50 species and is one of the most popular genera of Zingiberaceae because of its attractive foliage, diverse and showy flowers, and sweet fragrance [2]. *Hedychium stenopetalum* C. Lodd., is a potentially one of the tallest of all *Hedychium* species growing anything up to 3 to 4 m tall with big, bold oblong hairy leaves 8–10 cm long. This herbaceous plant has spikes of ghost white flowers with faint greenish-yellow tint to the base of the labellum. The stamens are white. The stems are about 30–40 cm long. The seeds are pale brown squarish shape 3-4 mm while the root is a thick pinky coloured spreading rhizome. *Hedychium stenopetalum* has a large natural range from north-eastern India to Guangxi province in China and down into northern Vietnam and Laos [3]. A glycoside, syringetin 3-rhamnoside was isolated from *H. stenopetalum* [4]. The major compounds in the rhizome essential oils of *H. stenopetalum* were *β*-pinene, linalool and 1,8-cineole [5].

*Hedychium coronarium* J. König is a perennial growing to 1.5 m (5 ft) by 1 m (3 ft 3in). It is in flower from Aug to October. The flowers are hermaphrodite (have both male and female organs). The seed is aromatic, carminative and stomachic [6]. The root is antirheumatic, excitant and tonic. The ground rhizome is used as a febrifuge. An essential oil from the roots is carminative and has anthelmintic indications [6]. Extracts of *H. coronarium* have been used for the treatment of inflammation, skin diseases, headache, and sharp pain due to rheumatism in traditional medicine [7]. The plant also possessed analgesic and neuropharmacological [8,9], anti-inflammatory [9], antimicrobial [10,11] and cytotoxic [11] activities. Phytochemical investigation revealed the isolation of labdane-type diterpenes coronarins G-I [7] as well as hedychicoronarin, peroxycoronarin D, 7β-hydroxycalcaratarin A and (*E*)-7β-hydroxy-6-oxo-labda-8(17),12-diene-15,16-dial which have potential anti-inflammatory benefits [12]. A diterpene named isocoronarin-D was also isolated from sample collected in Nepal [13]. In addition, a diterpenoid Galanal B, known for the regulation of blood glucose levels [14], benzoyl eugenol and ethoxy coronarin D [15], cytotoxic coronarin D [16], cytotoxic diterpenoids and diarylheptanoid [17] and hepatoprotective constituents [18] were the other notable compounds present in the plant. The chemical constituents of essential oils of various parts of *H. coronarium* have been documented with many chemotypic forms [19–34].

*Hedychium ellipticum* Buch.-Ham. ex Smith., is a fully hardy perennial semi-evergreen rhizome with cream and white flowers in late Summer, early Autumn and mid Summer. It grows well in semi-shade, and prefers high levels of water. The flowers are arranged in a umbelliform cyme inflorescence with orange pollen [35]. The methanolic extracts of *H. ellipticum* exhibited potent inhibitory action to the biosynthesis of leukotriene B (4) in bovine polymorphonuclear leukocytes with IC_50_ of 20 μg/mL [35]. The major components of essential oil of *H. ellipticum* [31] were 1,8-cineole (33.0%), sabinene (22.2%), terpin-4-ol (14.3%), *γ*-terpinene (5.3%) and *β*-caryophyllene (5.6%).

*Hedychium flavum* Roxb., is a pseudostems plant that grows up to 1.5-2 m. The leaves are with yellow flowers. Flowering takes place between August and September [3]. It has younger tender shoots and is used as vegetable and as food flavouring agent [36]. The authors are unaware of any biological effect and the chemical compounds isolated from this plant. However, Moellenbeck *et al.* [37] reported that the major components in *H. flavum* analysed from Madagascar essential oils were *β*-pinene (50%) and *β*-caryophyllene (27%). In contrast, the major constituents found in Turkish *H. flavum* essential oil [38] were *β*-pinene (23.7%), 1,8-cineole (28.3%), *α*-pinene (12.1%) and linalool (8.9%).

## Results and discussion

Additional file 1: Table S1 indicates the identities and the percentage compositions of compounds present in the *Hedychium* species. The identified compounds represent 95.3%-99.1% of the total oils content. *α*-Pinene (52.5% and 5.0% respectively) and *β*-pinene (31.8% and 12.3% respectively) were common to the leaf and root oils of *H. stenopetalum*. The root oil contained quantitative amount of linalool (45.2%) and (*E*)-nerolidol (8.7%) which were present in the leaf in much lower amounts (0.5% and 1.3% respectively). 1,8-Cineole, the main compound of a previous study [5] was not identified in the leaf oil but present in much lower quantity (1%) in the root oil. Though linalool and *β*-pinene as seen in the present study were of significant quantity in the precious work [5], the high content of *α*-pinene in our oil samples confer some quantitative and qualitative variations between results from Vietnam and elsewhere.

*α*-Pinene (7.6%, 10.4% and 4.3% respectively) and *β*-pinene (22.5%, 11.2% and 21.8% respectively) were the common compounds in the leaf, stem and root oils of *H. flavum. α*-Humulene (15.7%), *β*-caryophyllene (10.4%), limonene (7.0%) and bornyl acetate (6.0%) were the additional compound in the leaf oil while the stem contained significant amounts of *α*-humulene (18.9%), *β*-caryophyllene (11.8%), *α*-selina-6-en-4-ol (7.4%) and (*E*)-nerolidol (7.0%). However, linalool (17.5%), 1,8-cineole (13.5%), linalyl propionate (7.7%) and *γ*-terpinene (5.3%) were the other compound identified in the root oil. The high contents of *α*-pinene, *β*-pinene, 1,8-cineole and *β*-caryophyllene makes the oil similar to previous reports [37,38] but differs due to its high contents of linalool, linalyl propionate, *α*-humulene, (*E*)-nerolidol and *α*-selina-6-en-4-ol that were not detected in the previous studies.

The major constituents identified in the leaf oil of *H. ellipticum* were 1,8-cineole (40.8%), *α*-pinene (18.3%) and *β*-pinene (11.0%) while the root oil contained large amounts of (*E*)-nerolidol (15.9%), *β*-pinene (11.8%), bornyl acetate (9.2%), sabinene (8.4%), *α*-selina-6-en-4-ol (6.2%) and *δ*-selinene (5.9%). The oil composition differs considerably from a previous study [31] due to the lower contents of 1,8-cineole, terpin-4-ol, *γ*-terpinene and *β*-caryophyllene [31].

*Hedychium coronarium* afforded oils whose major constituents were *β*-pinene (20.0%), linalool (15.8%), *α*-pinene (10.1%), 1,8-cineole (10.7%) and *α*-terpineol (8.6%) in the leaf while the root consists mainly of *β*-pinene (23.6%), *α*-humulene (17.1%), *β*-caryophyllene (13.0%), *α*-pinene (6.9%) and elemol (6.9%). The volatile constituents of the various parts of *H. coronarium* from other parts of the world have been reported [19–34]. Although ubiquitous monoterpenes and sesquiterpeens were the main components of these oils, the identities of these compounds differed from one another. This led to the delineation of various chemotypic forms of the essential oils of *H. coronarium*. The compositional pattern of the leaf oil (*β*-pinene, linalool, *α*-pinene, 1,8-cineole) and the root (*β*-pinene, *β*-caryophyllene, *α*-humulene) in this study seems to be new chemotypic forms of essential oil of the plant when compared with previous studies (Additional file 2: Table S2).

The five chemotypes so far reported in the literature for the leaves oil were; *α*-pinene, *β*-pinene, 1,8-cineole, *β*-caryophyllene; *β*-pinene, *α*-pinene, 1,8-cineole, *γ*-elemene; *β*-*trans* ocimenone, linalool, 1,8-cineole; *β*-caryophyllene, caryophyllene oxide, *β*-pinene; and caryophyllene oxide, *β*-caryophyllene, caryophylladienol I. However, the four chemotypic forms of the rhizome oils include 1,8-cineole, *β*-pinene, *α*-terpineol, *α*-pinene; *trans-m*-mentha-2,8-diene, linalool, *α*-terpineol; *α*-muurolol, *α*-terpineol, 1, 8-cineole; and linalool, limonene, *trans*-meta-mentha,2,8diene. In addition, the flower oils also comprised of four main chemotypes namely linalool, methyl benzoate, *cis*-jasmone, eugenol; myrcenol, linalool, caryophyllene; *β*-pinene, 1,8-cineole, sabinene; and (*E*)-*β*-ocimene, linalool, 1, 8-cineole.

Several compounds such as sabinene, *trans-m*-mentha-2,8-diene, *β*-terpineol, *β*-*trans-*ocimenone, myrcenol, *cis*-jasmone, eugenol, jasmin lactone, methyl jasmonate, methyl-*epi*-jasmonate, indole, nitriles, oximes, *α*-muurolol, benzyl benzoate, phenol 2-methoxy-4 (1- propenyl), *α*-muurolene, decane, 2, 6-di-tert-butyl-4-methyl phenol, naphthalene, methylnaphthlene and 2-*β*-farnesene which were the characteristic compounds of previous investigated oil samples of *H. coronarium* were not detected in the present oil sample.

### Experimental

#### Plants collection

Leaf and roots of *H. stenopetalum* were collected from Mộc Châu District, Sơn La Province, Vietnam, in August 2011, while the leaf and roots of *H. coronarium* as well as the leaf, stems and roots of *H. flavum* were obtained from Bát Xát District, Lào Cai Province, Vietnam, in September 2011. The leaf and and roots of *H. ellipticum* were collected from Pù Mát National Park, Nghệ An, Province, in September 2011. Voucher specimens BVT 09, BVT 22, BVT 30 and BVT 25, respectively have been deposited at the Botany Museum, Institute of Ecology and Biological Resources, Vietnam Academy of Science and Technology. Plant samples were air-dried prior to extraction.

#### Extraction of the oils

Plant samples (0.5 kg each) were shredded and their oils were obtained by hydrodistillation for 3 h at normal pressure, according to the Vietnamese Pharmacopoeia [39]. The plant samples yielded essential oils as follows: 0.31 and 0.42% (v/w*; H. stenopetalum*; leaf and root respectively), 0.25 and 0.43% (v/w; *H. coronarium*; leaf and root respectively); 0.22, 0.23 and 0.37% (v/w; *H. flavum*; leaf, stem and root respectively), 0.23 and 0.31% (v/w*; H. ellipticum*; leaf and root respectively), calculated on a dry weight basis. Oil samples were light yellow coloured.

#### Analysis of the oils

Gas chromatography (GC) analysis was performed on an Agilent Technologies HP 6890 Plus Gas chromatograph equipped with a FID and fitted with HP-5MS column (30 m × 0.25 mm, film thickness 0.25 μm, Agilent Technology). The analytical conditions were: carrier gas H_2_ (1 mL/min), injector temperature (PTV) 250°C, detector temperature 260°C, column temperature programmed from 60°C (2 min hold) to 220°C (10 min hold) at 4°C/min. Samples were injected by splitting and the split ratio was 10:1. The volume injected was 1.0 μL. Inlet pressure was 6.1 kPa. Each sample was analyzed thrice. The relative amounts of individual components were calculated based on the GC peak area (FID response) without using correction factors.

An Agilent Technologies HP 6890 N Plus Chromatograph fitted with a fused silica capillary HP-5 MS column (30 m × 0.25 mm, film thickness 0.25 μm) and interfaced with a mass spectrometer HP 5973 MSD was used for the GC/MS analysis, under the same conditions as those used for GC analysis. The conditions were the same as described above with He (1 mL/min) as carrier gas. The MS conditions were as follows: ionization voltage 70 eV; emission current 40 mA; acquisitions scan mass range of 35–350 amu at a sampling rate of 1.0 scan/s.

#### Identification of constituents

The identification of constituents was performed on the basis of retention indices (RI) determined with reference to a homologous series of *n*-alkanes, under identical experimental conditions, co-injection with standards (Sigma-Aldrich, St. Louis, MO, USA) or known essential oil constituents, MS library search (NIST 08 and Wiley 9^th^ Version), and by comparing with MS literature data [40,41].

## Conclusion

For the first time, the compositions of the leaf and root essential oil of the Vietnamese grown *Hedychium stenopetalum, Hedychium coronarium* and *Hedychium ellipticum* as well as the leaf, stem and root of *Hedychium flavum* were elucidated. Although, ubiquitous terpenes were identified in the sample, the composition pattern was found to be different from the same species previously studied from other parts of the world. The quantitative composition and the relative proportions of the oil components are widely influenced by the factors such as nature and age of the plant genotype, ontogenic development, environmental and growing conditions, handling procedures e.t.c. These may largely be responsible for the observed compositional variations between the studied oil samples and previous results elsewhere.

## Additional files

Additional file 1: Table S1. Percentage compositions of compounds identified in *Hedychium* oils ^a^Standard deviation (SD) values were not included because there were no significant difference between values; ^b^Elution order on HP-5 MS column; ^c^Retention indices on HP-5MS column; ^d^Literature retention indices; ^e^Correct isomer not identified; Tr, trace amount < 0.1%; − Not identified. Additional file 2: Table S2. Major constituents of essential oils of *Hedychium coronarium* from literature. -Not known.
