# Supplementary material for: Composition of essential oils of four Hedychium species from Vietnam
Source: Chem Cent J. 2014 Aug 28;8:54. doi: 10.1186/s13065-014-0054-3 (PMC4161828; doi:10.1186/s13065-014-0054-3)
Supplement: Additional file 1: Table S1. — Percentage compositions of compounds identified in Hedychium oils aStandard deviation (SD) values were not included because there were no significant difference between values; bElution order on HP-5 MS column; cRetention indices on HP-5MS column; dLiterature retention indices; eCorrect isomer not identified; Tr, trace amount < 0.1%; − Not identified. [file 13065_2014_54_MOESM1_ESM.doc]

**Additional file 1: Table S1. Percentage compositions of compounds identified in *Hedychium* oils** a

| **Compounds^b^** | **RI^c^** | **RI^d^** | ***H. stenopetalum*** | | ***H. coronarium*** | | ***H. flavum*** | | | ***H. ellipticum*** | |
| --- | --- | --- | --- | --- | --- | --- | --- | --- | --- | --- | --- |
|  |  |  | **Leaf** | **Root** | **Leaf** | **Root** | **Leaf** | **Stem** | **Root** | **Root** | **Leaf** |
| *α*-Thujene | 930 | 924 | 0.2 | 0.1 | 0.3 | 1.7 | 0.8 | - | 0.2 | 0.3 | - |
| *α*-Pinene | 939 | 932 | 52.5 | 5.0 | 10.1 | 6.9 | 7.6 | 10.4 | 4.3 | 18.3 | 1.1 |
| Camphene | 953 | 946 | 0.4 | 0.7 | 1.6 | 1.3 | 2.6 | - | 0.7 | 1.2 | 1.8 |
| Verbenene | 962 | 961 | - | - | 0.1 | - | - | - | - | - | - |
| Sabinene | 976 | 969 | - | - | - | - | - | 0.5 | 0.3 | - | 8.4 |
| *β*-Pinene | 980 | 976 | 31.8 | 12.3 | 20.0 | 23.6 | 22.5 | 11.2 | 21.8 | 11.0 | 11.8 |
| *β*-Myrcene | 990 | 988 | 0.6 | 0.3 | 0.4 | 1.4 | 0.9 | - | 0.5 | 0.9 | 0.6 |
| *α*-Phellandrene | 1006 | 1002 | 0.9 | 0.9 | 0.6 | 0.1 | 0.2 | 0.5 | 1.3 | 0.5 | 0.2 |
| *δ*-3-Carene | 1011 | 1008 | - | 0.3 | 0.2 | - | - | - | 0.2 | 0.1 | - |
| *α*-Terpinene | 1017 | 1014 | 0.5 | - | 0.2 | 1.5 | 1.5 | 1.7 | 0.6 | 0.2 | 0.4 |
| *o*-Cymene | 1024 | 1022 | - | 2.2 | - | - | - | - | - | - | - |
| *p*-Cymene | 1026 | 1020 | 0.4 | - | 6.3 | - | - | - | 1.4 | - | - |
| Limonene | 1032 | 1024 | 1.1 | 1.4 | - | 3.8 | 7.0 | 0.5 | 1.6 | - | 1.9 |
| 1,8-Cineole | 1034 | 1030 | **-** | 1.0 | 10.7 | **-** | **-** | 0.2 | 13.5 | 40.8 | 3.5 |
| (*Z*)-*β*-Ocimene | 1043 | 1032 | - | - | - | 0.1 | - | - | - | - | - |
| (*E*)-*β*-Ocimene | 1052 | 1044 | 1.7 | 0.1 | 0.1 | 0.3 | - | - | 0.1 | 0.1 | - |
| *γ*-Terpinene | 1061 | 1054 | 1.5 | 0.2 | 4.7 | 0.9 | 0.4 | - | 5.3 | 0.6 | 0.9 |
| Linalool oxide ^e^ | 1076 | 1084 | - | 4.5 | - | - | - | - | 0.1 | 0.1 | - |
| *α*-Terpinolene | 1090 | 1086 | 0.6 | - | - | 0.4 | 0.3 | - | 0.4 | 0.5 | 0.3 |
| Methyl benzoate | 1184 | 1088 | - | - | 0.1 | **-** | **-** | **-** | - | **-** | **-** |
| Linalool | 1093 | 1095 | 0.5 | 45.2 | 15.8 | 0.1 | 0.3 | **-** | 17.5 | 3.7 | 0.7 |
| *trans*-Sabinene hydrate | 1100 | 1098 | - | - | - | 0.1 | - | - | 0.1 | 0.1 | 0.1 |
| *α*-Fenchyl alcohol | 1121 | 1118 | - | 0.6 | 0.5 | - | - | - | 0.1 | 0.1 | - |
| *allo*-Ocimene | 1128 | 1128 | - | - | 0.5 | - | - | - | - | - | - |
| *trans-*Pinocarveol | 1139 | 1135 | - | - | - | - | - | - | 0.3 | - | - |
| *trans*-Sabinol | 1140 | 1137 | - | 0.2 | - | - | - | - | - | - | - |
| Camphor | 1145 | 1141 | - | 0.2 | 0.3 | - | - | - | 0.4 | 1.2 | - |
| Pinocarvone | 1165 | 1160 | - | 1.1 | 2.0 | - | - | - | - | - | - |
| Borneol | 1167 | 1165 | 0.7 | 2.6 | 1.3 | 0.3 | 0.6 | - | 2.0 | 3.2 | 0.7 |
| Terpinene-4-ol | 1177 | 1174 | 0.9 | 2.5 | 2.4 | 0.4 | 0.3 | - | 2.5 | 4.5 | 0.8 |
| *α*-Thujenal | 1185 | 1182 | - | - | 0.2 | - | - | - | - | - | - |
| *α*-Terpineol | 1189 | 1187 | - | 3.7 | 8.6 | 0.1 | 0.3 | - | - | 5.1 | - |
| Myrtenol | 1194 | 1194 | - | 0.8 | - | - | - | - | 0.3 | - | - |
| Verbenone | 1205 | 1204 | - | - | 0.2 | - | - | - | - | 0.1 | - |
| Myrtenal | 1209 | 1195 | - | - | - | 0.1 | - | - | - | 0.3 | 0.1 |
| *trans*-Pulegol | 1214 | 1213 | - | - | - | - | - | - | - | - | 2.1 |
| *trans-*Carveol | 1217 | 1215 | - | 0.2 | 0.2 | - | - | - | - | 0.1 | - |
| *β*-Citronellol | 1222 | 1223 | - | - | - | - | - | - | 0.1 | - | - |
| Geraniol | 1253 | 1249 | - | 0.6 | 0.1 | 0.1 | - | - | 0.1 | 0.2 | - |
| (*E*)-Cinnamaldehyde | 1266 | 1267 | - | 1.5 | - | - | - | - | - | - | - |
| Bornyl acetate | 1284 | 1287 | 0.3 | 0.7 | 0.1 | 1.1 | 6.0 | 1.8 | 0.2 | - | 9.2 |
| *p*-Cymen-7-ol | 1289 | 1289 | - | - | - | - | - | - | - | 0.1 | - |
| Thymol | 1290 | 1289 | - | - | - | - | - | - | 0.1 | - | - |
| Carvacrol | 1300 | 1298 | - | - | 0.4 | - | - | - | - | - | - |
| *δ*-Terpinenyl acetate | 1318 | 1316 | - | - | - | - | - | - | 1.2 | - | - |
| Linalool propaonate | 1330 | 1334 | - | - | - | - | - | - | 7.7 | - | 0.1 |
| Bicycloelemene | 1337 | 1338 | - | - | - | 0.1 | - | - | - | - | - |
| *δ*-Elemene | 1340 | 1335 | - | - | - | 0.1 | - | - | - | - | 1.4 |
| *α*-Cubebene | 1351 | 1345 | - | - | - | - | - | - | - | 0.1 | - |
| Neryl acetate | 1362 | 1359 | - | - | - | 3.2 | - | - | - | - | - |
| Isoledene | 1372 | 1374 | - | - | 0.1 | - | - | - | - | - | - |
| *α*-Copaene | 1377 | 1374 | - | 0.2 | - | - | - | - | - | - | 0.2 |
| Geranyl acetate | 1381 | 1379 | - | - | - | - | - | - | - | - | 0.2 |
| *β*-Cubebene | 1388 | 1387 | - | - | - | - | - | - | - | - | 0.3 |
| *β*-Elemene | 1391 | 1389 | - | - | - | 0.7 | 0.6 | 0.7 | - | - | 0.5 |
| Methyl eugenol | 1407 | 1403 | - | - | Tr | - | - | - | 0.2 | 0.1 | - |
| *α*-Cedrene | 1409 | 1410 | - | - | 0.8 | - | 1.8 | 2.8 | 0.2 | - | - |
| *α*-Gurjunene | 1412 | 1409 | - | - | - | - | - | - | - | - | 0.7 |
| (*E*)-*β*-Damascone | 1415 | 1413 | - | - | - | - | - | - | 0.1 | - | - |
| *β*-Caryophyllene | 1419 | 1417 | 0.5 | - | 0.1 | 13.0 | 10.4 | 11.8 | 0.3 | 0.6 | 1.7 |
| *trans*-*α*-Bergamotene | 1436 | 1435 | - | - | - | - | 0.1 | - | 0.1 | - | 0.1 |
| *γ*-Elemene | 1437 | 1434 | - | - | - | 0.2 | 0.7 | 1.0 | 0.1 | - | 0.8 |
| Aromadendrene | 1441 | 1439 | - | - | - | 0.1 | 0.4 | - | - | - | 0.6 |
| *trans*-Cinnamyl acetate | 1445 | 1443 | - | 0.1 | - | - | - | - | - | - | - |
| 3,7-Guaiadiene | 1450 | 1444 | - | - | - | - | 0.4 | 0.5 | - | - | 0.4 |
| *α*-Humulene | 1454 | 1452 | 0.1 | **-** | **-** | 17.1 | 15.7 | 18.9 | **-** | 0.1 | 2.2 |
| *γ*-Gurjunene | 1473 | 1475 | - | - | - | - | - | - | 0.2 | - | - |
| *ar*-Curcumene | 1478 | 1479 | - | - | - | - | 2.5 | 3.1 | 0.1 | 0.2 | - |
| *γ*-Curcumene | 1483 | 1481 | - | 0.2 | - | 0.3 | - | - | 0.2 | 0.2 | 0.9 |
| Germacrene D | 1485 | 1484 | - | - | - | 0.9 | - | 0.5 | - | - | 0.3 |
| *α*-Amorphene | 1485 | 1483 | - | - | 0.1 | - | - | - | 0.3 | - | - |
| *β*-Selinene | 1486 | 1486 | - | - | 0.1 | 0.2 | - | - | - | - | - |
| *δ*-Selinene | 1493 | 1492 | - | - | - | 0.2 | 1.8 | - | 0.2 | - | 5.9 |
| *α*-Zingiberene | 1494 | 1493 | - | - | - | - | 0.2 | - | - | - | - |
| Valencene | 1496 | 1496 | - | 0.3 | - | - | - | - | - | - | - |
| Bicyclogermacrene | 1500 | 1500 | - | - | - | 1.3 | 0.9 | 1.0 | - | 0.4 | - |
| Epizonarene | 1502 | 1501 | - | - | - | - | 0.4 | 0.7 | - | - | - |
| *β*-Bisabolene | 1506 | 1505 | - | - | 0.1 | - | - | 1.0 | - | 0.1 | - |
| (*E,E*)-*α*-Farnesene | 1508 | 1505 | - | - | 0.1 | 0.1 | - | - | - | - | 0.6 |
| *γ*-Cadinene | 1514 | 1513 | - | - | 0.5 | - | - | - | - | - | - |
| *δ*-Cadinene | 1525 | 1522 | 0.8 | 0.1 | 0.9 | 0.4 | 0.4 | 3.6 | - | - | 0.9 |
| *cis*-Calamenene | 1530 | 1528 | - | - | - | - | - | - | - | - | 0.4 |
| *α*-Calacorene | 1546 | 1544 | - | - | - | - | 0.1 | - | - | - | 0.2 |
| Elemol | 1550 | 1548 | - | - | - | 6.9 | - | - | 0.1 | 0.3 | - |
| (*E*)-Nerolidol | 1563 | 1561 | 1.3 | 8.7 | 3.0 | 1.7 | 2.2 | 7.0 | 4.6 | 0.5 | 15.9 |
| Spathulenol | 1578 | 1577 | - | - | 0.1 | - | 1.1 | 2.6 | 0.2 | 0.1 | - |
| Caryophyllene oxide | 1583 | 1581 | 0.7 | - | 0.3 | 1.3 | 1.1 | 2.3 | 0.5 | 1.3 | 2.0 |
| Viridiflorol | 1593 | 1592 | - | - | - | 0.5 | - | - | - | - | - |
| Guaiol | 1601 | 1600 | - | - | - | - | - | - | 1.1 | - | 1.0 |
| Ledol | 1603 | 1602 | - | - | 0.1 | - | - | - | - | - | 1.4 |
| *γ*-Eudesmol | 1630 | 1630 | 0.1 | 0.2 | 0.3 | 2.6 | - | - | 0.8 | - | - |
| Aromadendrene epoxide | 1640 | 1639 | - | - | - | 0.1 | - | - | - | - | - |
| *τ*-Muurolol | 1646 | 1640 | - | - | 1.4 | - | 1.6 | 1.6 | - | - | 3.3 |
| *α*-Selina-6-en-4-ol | 1648 | 1650 | - | - | - | - | 2.8 | 7.4 | - | - | 6.2 |
| *α*-Cadinol | 1654 | 1652 | - | 0.4 | 0.7 | - | - | 3.9 | - | 0.2 | 2.2 |
| *α*-Eudesmol | 1659 | 1652 | - | - | - | 2.5 | - | - | - | - | - |
| Farnesol ^e^ | 1718 | 1712 | - | - | - | - | - | - | - | - | 1.1 |
| Octadecane | 1800 | 1800 | - | - | - | - | - | - | 0.1 | - | - |
| Dibutyl phthalate | 1957 | 1940 | - | - | - | - | - | 1.7 | - | - | - |
| Eicosane | 2000 | 2000 | - | - | - | - | - | - | 0.9 | - | - |
| Heneicosane | 2100 | 2100 | - | - | - | - | - | - | 0.1 | - | - |
| Phytol | 2125 | 1942 | - | - | - | - | - | - | - | - | 0.7 |
| **Total** | | | **98.1** | **99.1** | **96.7** | **98.9** | **97.5** | **98.1** | **99.3** | **97.5** | **96.8** |
| **Monoterpene hydrocarbons** | | | **92.2** | **23.5** | **45.1** | **42.0** | **44.8** | **24.8** | **39.7** | **33.7** | **27.4** |
| **Oxygenated monoterpenes** | | | **2.4** | **65.5** | **42.8** | **5.6** | **7.5** | **2.0** | **49.4** | **59.7** | **17.5** |
| **Sesquiterpene hydrocarbons** | | | **1.4** | **0.8** | **2.8** | **34.7** | **36.4** | **44.6** | **1.8** | **1.7** | **18.1** |
| **Oxygenated sesquiterpenes** | | | **2.1** | **9.3** | **5.7** | **15.6** | **8.8** | **24.8** | **7.3** | **2.4** | **33.1** |
| **Diterpenes** | | | **-** | **-** | **-** | **-** | **-** | **-** | **-** | **-** | **0.7** |
| **Fatty acids** | | | **-** | **-** | **-** | **-** | **-** | **-** | **1.1** | **-** | **-** |
| **Others** | | | **-** | **-** | **0.1** | **-** | **-** | **1.7** | **-** | **-** | **-** |

^a^ Standard deviation (SD) values were not included because there were no significant difference between values; ^b^ Elution order on HP-5 MS column; ^c^ Retention indices on HP-5MS column; ^d^  Literature retention indices; ^e^ Correct isomer not identified; Tr, trace amount < 0.1%; - Not identified.
