# Supplementary material for: Composition of essential oils of four Hedychium species from Vietnam
Source: Chem Cent J. 2014 Aug 28;8:54. doi: 10.1186/s13065-014-0054-3 (PMC4161828; doi:10.1186/s13065-014-0054-3)
Supplement: Additional file 2: Table S2. — Major constituents of essential oils of Hedychium coronarium from literature. -Not known. [file 13065_2014_54_MOESM2_ESM.docx]

**Additional file 2: Table S2. Major constituents of essential oils of *Hedychium coronarium* from literature**

| Origin | Biological activities | Plant Parts | Major constituents | References |
| --- | --- | --- | --- | --- |
| - | Antifungal and antibacterial | Fresh rhizome | 1,8-cineole (41.42%), *β*-pinene (10.39%), α-terpineol (8.8%) | 19 |
| - | Antifungal and antibacterial | Dried rhizome | 1,8-cineole (37.44%), *β*-pinene (17.4%), *α*-terpineol (6.7%) | 19 |
| Japan | - | Flower | linalol, methyl jasmonate, eugenols, cis-jasmone, *β*-ionone, lactones | 20 |
| Cuba | - | Flower | (*E*)-*β*-ocimene (28.7%), linalool (19.3%), 1,8-cineole (14.5%) | 21 |
| China | - | - | *β*-pinene (21.73%),1,8-cineole (11.8%), sabinene (11.32%), | 22 |
| Japan |  | Flower | linalool, methyl benzoate, *cis*-jasmone, eugenol, (E)-isoeugenol, jasmin lactone, methyl jasmonate, methyl *epi-*jasmonate, indole, nitriles and oximes | 23 |
| China | - | Flower | myrcenol, linalool, *β*-caryophyllene, *β*-terpineol, benzyl benzoate | 24 |
| Brazil | - | Leaf | *β*-caryophyllene (43.0%), caryophyllene oxide (12.1%), *β*-pinene (11.6%,) | 25 |
| ‘’ | - | Rhizome | 1,8-cineole (34.8%), *β*-pinene (16.7%), *α*-terpineol (13.1%) | ‘’ |
| India | Antibacterial | Rhizome | linalool (29.3 %), limonene (20.3 %), *trans*-m-mentha,2,8diene (12.9 %), *γ*-terpinene (8.9 %) | 26 |
| Polynesia | - | Rhizome | *β*-pinene (24.8%) and 1, 8-cineole (40.2%) | 27 |
| India | Antibacterial | Rhizome | 1,8-cineole (48.7%) | 28 |
| Mauritius | - | Rhizome | *α*-muurolol (16.8%), *α*-terpineol (15.9%), 1, 8-cineole (11.2%), | 29 |
| Fijian | - | Rhizome | *α*-pinene (10.6%), *β*-pinene (31.4%), 1,8-cineole (55.9%) | 30 |
| Fijian | - | Leaf | *β*-pinene (53.6%), *α*-pinene (20.9%), 1,8-cineole (11.9%), *β*-caryophyllene (17.7%) | 30 |
| India | Antioxidant and antibacterial | Rhizome | trans-m-mentha-2,8-diene (25.2%), linalool (21.7%), *α*-terpineol (10.9%) | 31 |
| China | Antioxidant | Rhizome | *β* -*trans* ocimenone (28.05%), linalool (18.52%), 1,8-cineole (11.35%) | 32 |
| China | Antimicrobial larvicidal and antioxidant | Leaf | *β*-pinene (33.9%), *α*-pinene (14.7%), 1,8-cineole (13.3%), *γ*-elemene (11.0%), carotol (9.1%) | 33 |
| ‘’ | Antimicrobial larvicidal andantioxidant | Rhizome | 1,8-cineole (37.3%), *β*-pinene (23.0%),  *α*-terpineol (10.4%) and *α*-pinene (9.9%) | ‘’ |
| Brazil | Anti-trypanocidal | Rhizome | 1,8-cineole (31.7%), coronarin E (14.1%), 𝛼-terpineol (12.0%), 𝛽-pinene (11.0%) | 34 |
| ‘’ | Anti-trypanocidal | Leaf | caryophyllene oxide (43.9%), *β-*caryophyllene (12.1%) and caryophylladienol I (7.7%) | 34 |

- Not known
